# Cultural aspects of primary healthcare in india: A case- based analysis

**DOI:** 10.1186/1447-056X-10-8

**Published:** 2011-06-16

**Authors:** Roger P Worthington, Anupriya Gogne

**Affiliations:** 1 School of Medicine, Keele University, City General Hospital, Newcastle Road, Stoke-on-Trent, ST4 6QG, UK; 2 School of Medicine, Vardhman Mahavir Medical College, Safdarjang Hospital, New Delhi - 110 029, India

## Abstract

Delivering quality primary care to large populations is always challenging, and that is certainly the case in India. While the sheer magnitude of patients can create difficulties, not all challenges are about logistics. Sometimes patient health-seeking behaviour leads to delays in obtaining medical help for reasons that have more to do with culture, social practice and religious belief. When primary care is accessed via busy state-run outpatient departments there is often little time for the physician to investigate causes behind a patient's condition, and these factors can adversely affect patient outcomes. We consider the case of a woman with somatic symptoms seemingly triggered by psychological stresses associated with social norms and familial cultural expectations. These expectations conflict with her personal and professional aspirations, and although she eventually receives psychiatric help and her problems are addressed, initially, psycho-social factors underlying her condition posed a hurdle in terms of accessing appropriate medical care. While for many people culture, belief and social norms exert a stabilising, positive influence, in situations where someone's personal expectations differ significantly from accepted social norms, individual autonomy can be directly challenged, and in which case, something has to give. The result of such challenges can negatively impact on health and well-being, and for patients with immature defence mechanisms for dealing with inner conflict, such an experience can be damaging and ensuing somatic disturbances are often difficult to treat. Patients with culture-bound symptoms are not uncommon within primary care in India or in other Asian countries and communities. We argue that such cases need to be properly understood if satisfactory patient outcomes are to be achieved. While some causes are structural, having to do with how healthcare is accessed and delivered, others are about cultural values, social practices and beliefs. We note how some young adult women are adversely affected and discuss some of the ethical issues that arise.

## Review

India is a country with a diverse range of cultures, ethnicities, religions and languages. While in many ways this is a source of richness and strength, cultural influences sometimes give rise to challenges in the context of managing commonly presenting illnesses. Physicians caring for patients expect to take account of psychological, social and environmental factors that underlie some of the problems with which patients present in general practice, particularly where there are concerns about mental health. But in cases where physical manifestations seem to stem from deep-seated influences relating to socio-cultural norms and expectations, some conditions can prove difficult to treat. In our view, interconnections between socio-cultural factors and health need to be better acknowledged and warrant exploration in the hope of making it easier to achieve best practice and improve patient outcomes. Against this background we consider a case from India involving a young woman who presents late with an underlying psychiatric disorder, paying particular attention to ethical, cultural and social aspects of her care.

## Background

As a nation India faces a number of challenges in trying to meet population needs for quality healthcare. For instance, in primary health clinics and state-run community hospitals the length of an average consultation is just a few minutes, which makes it hard to take account of underlying socio-economic and psycho-social factors. The short consultation means that it is difficult to investigate adverse factors impacting on patients' physical and psychological well-being. However, on the positive side, primary healthcare offered by city and district hospitals and by rural primary health centres generally succeed in offering basic treatment with no cost to the patient. The focus in primary care clinics is usually on immunization, treatment of common illnesses, prevention of malnutrition, and providing pregnancy, childbirth and postnatal care; patients needing specialised care (and/or having more complicated illnesses) are referred to secondary and tertiary care centres, which may have district, state or national teaching hospital status.

GPs tend to have well-established connections with such centres enabling them to make suitable referrals, especially in urban areas; patients in turn have long-standing connections with GPs, sometimes extending over generations. Broadly speaking, healthcare in India is divided between private and state-funded and between rural and urban centres, and these divergent limbs form part of a complex system that tries to cater for the needs of a vast population. On the one hand it succeeds in catering for large numbers of patients providing basic care for uncomplicated ailments; on the other hand compromises have to be made regarding quality of care, especially when treating illnesses that demand resource-intensive standards of care.

Some people have the chance to access quality, private insurance-based healthcare, but others are excluded due to lack of affordability. Imbalances arise between the private and state-funded health sectors, and these are both significant and growing [1]. While the Indian system relies on a mix of primary health village centres and government hospitals to provide free medical care for the general population, private hospitals cater more for urban, higher socio-economic strata in society. The public system seeks to make healthcare accessible to all sectors of the population and it was structured with this in mind. However, the system does not always function in the way that was originally intended due to problems such as poor standards of literacy, overt political and religious influences, an ever-expanding population, and poor doctor to patient ratios.

These factors can combine to form a vicious circle, and the healthcare system often lacks the necessary resources to enable proper provision of inpatient facilities, including basic essential medical equipment or help with transport for patients coming from more remote geographical locations. Patients from these areas experience additional barriers in terms of accessing quality, affordable, local care. Hindrances have to do with politics as well as geography, and on account of relatively low standards of education there is often a general lack of awareness about family planning.

### Culture, Belief and Health

Against this background we consider the role played by culture and belief and how they can impact on patient outcomes. Belief systems and moral values are intrinsic to human life, and for many people cultural and religious considerations exert strong, positive influences on their lives. But norms bound by culture and belief can also negatively impact on people in terms of mental and physical well-being. Culture-bound syndromes are not uncommon within primary care in India and Asian communities more generally, with cases arising that display psychiatric and associated somatic symptoms [2]. Recognising that there is an element of controversy surrounding the diagnosis, [3] an example we wish to consider is that of dissociative trance or possession-like state, most commonly encountered amongst young adult women. Dissociative trance or possession states capture the essence of the problems we are addressing, and we offer the following case as a way of exploring them further.

### Case Study

'S' is a 23 year-old female who presents with episodes of anxiety, accompanied by feelings of impending doom, shortness of breath, palpitations, and loss of sensation in her limbs lasting for 15-20 minutes. Symptoms are accompanied by a shift in consciousness whereby 'ancestral spirits' appear to take control over her body and personal identity. This experience is accompanied by violent behaviour, a change in voice and irrelevant speech content, as well as general weaknesses, body aches and decreased appetite. Anxieties appear to result in somatic symptoms with autonomic instability. However, S is reluctant to seek psychiatric help, partly because of the stigma attached to this kind of therapy.

Family levels of education range from illiteracy to having full secondary education; the family is closely-knit and conforms to conventional societal norms. S is well-educated and a graduate with ambitious plans for further study; however, these are interrupted when she becomes engaged as part of an arranged marriage. She experiences a number of problems and has no recollection of episodes involving 'possession and dissociative trance'; eventually a decision is made to consult the local religious healer, whom the family has been seeing for generations. S is taken to a temple where rituals are carried out to 'drive out spirits from her body'. Her symptoms improve but only for a matter of days. The family eventually seeks help and advice from the GP for S's abnormal behaviour, and the GP makes a referral to a local psychiatrist. S and her family are open to psychotherapy, although it requires motivation and persuasion in order to try and break the cycle of events; in total, the delay in seeking qualified help amounts to six to eight months, largely by reason of family beliefs and S's lack of insight into her illness.

Eventually she is treated and her psychological stressors are identified, namely that S was unwilling to get married and felt unable to convey this to her parents or express her desire to do further studies; S felt it would be disrespectful towards her parents to talk openly about such matters. At the consultation it was thought that subconsciously she was using denial and dissociation to cope with stresses arising from her internal conflict. She is eventually admitted to a local hospital for psychiatric observation and 'distraction therapy'.

Gradually, psycho-educative sessions involving both patient and the family are undertaken to clear away apparent misconceptions about healing rituals, while at the same time stressing the importance of seeking out and complying with professional treatment and advice. Without being disrespectful to values and sentiments of the parents, the focus gradually shifts towards S's long-term professional ambition. The advice given is for her not to marry until she feels that she is ready.

Upon further psychiatric evaluation S is found to have 'histrionic personality traits and immature maladaptive defence mechanisms' for coping with family rules based on strict societal norms and expectations. After a week of observation as an inpatient no further episodes of abnormal behaviour are seen. Subsequently S receives regular psychotherapy as an outpatient and makes a steady improvement.

## Discussion

Culture and religion played a significant role in the course of her illness from original diagnosis through to treatment and eventual prognosis. To understand the aetiology of her condition and navigate some of the roadblocks to accessing timely medical care it is important to note the effect of culture and religious belief, particularly regarding 'ancestral spirits' (however that term is understood). Such manifestations are not uncommon, [4] and presenting symptoms are often caused by maladaptive responses, especially in younger women.

Such family pressures need not be negative in their effect, and family values and ideals inculcated over generations can exert positive influences by providing crucial support. The role of family should not be undervalued, and the concept of joint, extended families is central to everyday living, providing economic as well as psycho-social stability. Family support is often vital to the recovery of patients, especially for those with advanced illnesses. Whole families sometimes make long trips to hospital with the patient in order to seek the necessary help, and outside the setting of public hospitals it can fall to the extended family to pay for treatment from their own private resources, which in the case of a young mother, could be with help from a grandparent.

It must be borne in mind that people with different cultural, religious and demographic profiles will not always react in the same way, and to ensure optimal care for patients such as S it is important to try and consider all determinants of physical and mental wellbeing. Delays in seeking professional help as well as issues of non-compliance once therapy is eventually started are commonly found in cases such as this, stemming from deeply held faith in religious healing and the associated rituals [5]. The trend towards finding healers first and then doctors covers all fields of medicine in India and beyond. It cannot be wrong to show respect for cultural traditions and belief, but if pursued without heed to possible harms that arise from *not *seeking timely professional help the situation could change. Blind adherence to conventional patterns of behaviour may not be ethically defensible if the consequences are harmful to the patient; therefore, judgment needs to be exercised in order to properly assess a situation such as the one we describe.

Key to S's case are social tensions between respecting rights to live in accordance with established cultural tradition and social norms, and respecting individual autonomy to make free, independent personal choices. Mutual incompatibility between these positions lies at the root of problems seen in this and similar cases. The pressure felt by S to conform to social and family values led her to abandon her career aspirations without having adequate coping mechanisms for dealing with the resultant inner conflict. S's family explain her condition by making reference to 'ancestral spirits', reflecting patterns of traditional belief and health seeking behaviours commonly associated with Indian cultural beliefs.

Old and new forms of belief in health and healing can comfortably co-exist, although in this case belief in cultural tradition means that spiritual healers are consulted *before *consideration is given to seeking out Western forms of medicine. While the delay did not help the patient, traditional forms of medicine and healing are nonetheless part of the web of everyday life. Just as it is naïve to suggest that modern allopathic medicine has an answer to every problem, so it is wrong to suppose that allopathic medicine always has to be a first line of defence. We suggest that having an open mind is the best and most appropriate response.

The issue here is not about lack of education, because articulate, well-educated people can still express preferences for traditional over modern forms of medicine; nor is it about prioritising one system of medicine over another. It is about finding an accommodation for a workable co-existence between different forms of medicine and dealing with conflicting attitudes, values and preferences. Furthermore, the situation we describe is not unlike that which is commonly found in China, where the two different types of medicine (traditional and allopathic) generally work in parallel, and have done for many years [6].

The role played by culture and religion in Indian culture is undeniable, but it must be remembered that cultural tradition and religious beliefs are not necessarily the same thing. For instance, people can describe themselves as belonging to a dominant culture but without subscribing to the tenets of the main associated religion. The converse can also be true, and someone can be religious without having any particular interest in local culture; furthermore, someone can be interested in spirituality but not organised religion. In short, culture, religion, spirituality and belief may or may not be coterminous, and these distinctions are worth bearing in mind. Here the issue is about respecting the role played by cultural values in people's lives and navigating a path between old and new modes of living in order to try and optimise patient outcomes.

### Women's health

The social aspiration of women is an important part of this discussion, and the case study highlights questions surrounding family values, arranged marriages, and rights to education. None of these issues should be viewed in isolation, and while the first two points are more symbolic of 'old' culture, the third is more about 21^st ^century social and political aspirations. Clearly, not all women experience mental health problems if they express preferences to uphold rights to individual autonomy over family values or enrol in higher education; that does not follow. However, in our case internalised social, familial pressure acted as a trigger for the onset of mental illness, causing the patient to present with a range of seemingly disconnected symptoms. Family belief, coupled with stigma leading to apprehension, commonly found in relation to people's attitude towards psychiatry, created obstacles in the path of our patient[7,8].

### Patient autonomy

In terms of the underlying ethics, patient autonomy in Indian culture may not play the same role that it does in the West; [9] sometimes the moral imperative is to be seen and treated more than presented with choices that may not exist (especially in publicly funded healthcare). This leads to an awkward if inescapable conclusion that in practical terms autonomy may mean different things in different cultural and geographic settings, or at least it can have different applications. There is limited scope within this paper to discuss these ethical tensions fully, and our case is not about patient autonomy in the customary sense of reaching joint decisions about clinical care. Rather, it is about broader relationships between personal autonomy, family and society. Indian society is multi-faceted, and the same applies to other Asian cultures, and while we do not merely seek to draw generalisable conclusions, we do want to highlight a set of phenomena that are not uncommon and that are not always well understood.

## Conclusion

In the primary care setting, even though time may be a scarce resource, it is important to be mindful of social and cultural factors that can negatively impact on patient well-being. The problems encountered in relation to patient S are not uncommon in India or in Asian culture more generally. The root cause of the difficulties we describe and the potential social remedies probably lie beyond the power of the family physician to solve because they are so deep-seated. However, they can and do affect day-to-day clinical practice, and we argue that such issues have a moral and a practical relevance that merits wider discussion and recognition.

We end with these key summary points:

1. If there is limited time for discussion about patient values and beliefs, this may present a real challenge, especially where socio-economic and cultural factors play a role.

2. Keep an open mind as to the cause of symptoms presented by patients within family medicine (especially in the case of young adult women).

3. Recognise that it may be beyond the power of the clinician to address underlying factors affecting patient well-being.

4. Different systems of medicine can work in parallel, and while spiritual healing has a long tradition, sole reliance on it sometimes leads patients to avoid seeking other forms of care.

5. Cultural values, spirituality, and religious belief do not necessarily concern one and the same thing; it is possible to be influenced by one but not others.

6. While patient autonomy is important it can lead to tension with cultural and social norms and satisfactory outcomes may have to be negotiated.

## Competing interests

The authors declare that they have no competing interests.

## Authors' contributions

RW planned the article and acted as first author; AG provided the case study and background information; both authors contributed to the final draft and have read and approved the final manuscript. RW and AG would like to thank Sinthuja Visahan (Keele University, UK) for help in researching references.

## Authors' information

RW lectures at Keele University School of Medicine (UK), where he leads on ethics and law. He is an Assistant (Adjunct) Professor of Medicine at Yale University (USA) and an Associate (Adjunct) Professor of Medical Ethics and Law at Bond University (Australia). Roger has a lifelong interest in Indian philosophy and culture.

AG graduated from Vardhman Mahavir Medical College in New Delhi (India), and she is starting psychiatric residency training at the State University of New York, Brooklyn (USA).
